# Completion of Eight *Gynostemma* BL. (Cucurbitaceae) Chloroplast Genomes: Characterization, Comparative Analysis, and Phylogenetic Relationships

**DOI:** 10.3389/fpls.2017.01583

**Published:** 2017-09-12

**Authors:** Xiao Zhang, Tao Zhou, Nazish Kanwal, Yuemei Zhao, Guoqing Bai, Guifang Zhao

**Affiliations:** ^1^Key Laboratory of Resource Biology and Biotechnology in Western China (Ministry of Education), College of Life Sciences, Northwest University Xi'an, China; ^2^College of Biopharmaceutical and Food Engineering, Shangluo University Shangluo, China; ^3^Xi'an Botanical Garden of Shaanxi Provence, Institute of Botany of Shaanxi Provence Xi'an, China

**Keywords:** *Gynostemma* BL., chloroplast genome, characterization, comparison, repeats, phylogeny

## Abstract

*Gynostemma* BL., belonging to the family Cucurbitaceae, is a genus containing 17 creeping herbaceous species mainly distributed in East Asia. It can be divided into two subgenera based on different fruit morphology. Herein, we report eight complete chloroplast genome sequences of the genus *Gynostemma*, which were obtained by Illumina paired-end sequencing, assembly, and annotation. The length of the eight complete cp genomes ranged from 157,576 bp (*G. pentaphyllum*) to 158,273 bp (*G. laxiflorum*). Each encoded 133 genes, including 87 protein-coding genes, 37 tRNA genes, eight rRNA genes, and one pseudogene. The four types of repeated sequences had been discovered and indicated that the repeated structure for species in the Subgen. *Triostellum* was greater than that for species in the Subgen. *Gynostemma*. The percentage of variation of the eight cp genomes in different regions were calculated, which demonstrated that the coding and inverted repeats regions were highly conserved. Phylogenetic analysis based on Bayesian inference and maximum likelihood methods strongly supported the phylogenetic position of the genus *Gynostemma* as a member of family Cucurbitaceae. The phylogenetic relationships among the eight species were clearly resolved using the complete cp genome sequences in this study. It will also provide potential molecular markers and candidate DNA barcodes for future studies and enrich the valuable complete cp genome resources of Cucurbitaceae.

## Introduction

*Gynostemma* BL., which belongs to the family Cucurbitaceae, is a genus containing 17 creeping herbaceous species mainly grown in moist mountains, forests, thickets, and streamside (Chen et al., 2011). According to different fruit morphology, all species are divided into 2 subgenera: Subgen. *Gynostemma* with berries and Subgen. *Triostellum* with capsules. The species of the former have a wide distribution in East Asia, especially in subtropical China, Japan, Myanmar, and India, whereas the latter are endemic to southern China (Chen, 1995). The most widespread species (*G. pentaphyllum*) and seven microspecies were studied in our current study, four of these (*G. pentaphyllum, G. longipes, G. pubescens*, and *G. burmanicum*) belong to Subgen. *Gynostemma*, and the remaining four (*G. cardiospermum, G. laxiflorum, G. caulopterum*, and *G. pentagynum*) are Subgen. *Triostellum* species.

In the past, most known studies of the genus *Gynostemma* mainly focused on extracting bioactive components (Yin et al., 2004), chemistry, or pharmacology (Razmovski-Naumovski et al., 2005; Tsai et al., 2010). As a traditional Chinese medicinal herb, *G. pentaphyllum* has a high content of saponin and dissociative amino acids. It is useful in clinical and medical science because of its hypoglycemic activity, anticancer effects, and immunity enhancement (Xie et al., 2010). In fact, most of the species from the genus *Gynostemma* are valuable in the production of medicine, whereas the differences lie in the amount of active ingredients (Liu et al., 2006). Currently, with the development and utilization of medicinal plants, the use of natural medicine by people is growing. Therefore, identification of wild species is particularly important. However, it is difficult to define a classification of species within the genus *Gynostemma* because of hybridization, and more transitional taxa were found in the wild. Thus, it is necessary to develop more genomic resources for population genetics and DNA barcodes for species identification within the genus *Gynostemma*.

Additionally, the few genetic studies on this genus was restricted to the development of molecular markers (Zhou et al., 2010; Liao et al., 2011), the identification and relationships among a minority of species, and the population genetics of single species (Wang et al., 2008a; Jiang et al., 2009). However, these studies only used a few specific fragments of DNA that may lead to an incomplete conclusion. Therefore, we prefer to use the whole genome, especially the complete chloroplast genome, to resolve the problems discussed herein.

The chloroplast (cp) is an important organelle found in green plants where photosynthesis and energy transformation occurs. It is inherited in a maternal manner in the majority of plants. Compared with the nuclear genome and mitochondria genome, the cp genome is smaller in size and is a circular double-stranded DNA molecule. Matrilineal inheritance allows stable transgene expression without gene contamination. Moreover, the cp genome has a moderate rate of nucleotide evolution, but shows a large difference in the rate of divergence between coding (CDS) and non-coding (CNS) regions. This makes the cp genome suitable for phylogenetic studies at different taxonomic levels (Li et al., 2013).

An increasing number of cp genomes of higher plants have been obtained since the first complete cp genome of *Nicotiana tabacum* was determined (Shinozaki et al., 1986; Li et al., 2016). However, only a few complete cp genomes of Cucurbitaceae have been reported, and even fewer *Gynostemma* species. In angiosperms, most cp genomes are made up of four parts: a pair of inverted repeats (IRa and IRb), one large single-copy region (LSC) and one small single-copy region (SSC) (Palmer, 1985). Because of the expansion and contractions of the IR regions, the size of cp genomes have ranged from 120–160 kb (Wang et al., 2008b). The comparative analysis of the complete cp genomes provided information on genome structure. It also played an important role in understanding the cp genome evolution, species identification, and phylogenetic relationships (Yang et al., 2016).

In the present study, the comparative analysis of eight complete *Gynostemma* cp genomes was conducted to explore the features and structural differentiation of the sequences, as well as enrich the valuable complete cp genome resources of the family Cucurbitaceae. The simple sequence repeats (SSRs), which are also known as microsatellites, could serve as potential molecular polymorphic markers for genetic diversity and genetic structure of *Gynostemma* populations in the future. Highly variable regions would provide candidate DNA barcodes for coming studies. This study reconstructed the phylogenetic relationship and verified the morphologic phylogenetic position of the genus *Gynostemma* in Cucurbitaceae. Furthermore, our study will contribute to further studies on the phylogenetic analysis within the genus *Gynostemma* and enhanced our profound understanding of the systematic evolution of Cucurbitaceae.

## Materials and methods

### Plant materials and DNA extraction

Fresh and healthy leaves were collected from adult plants for eight species (Table 1). Voucher specimens were deposited in the Key Laboratory of Resource Biology and Biotechnology (Shaanxi, China). The chloroplast genomic DNA of *G. pentaphyllum* was isolated from fresh leaves using the density gradient centrifugation method (Sandbrink et al., 1989) and the CTAB extraction method, and each of the total genomic DNA for the seven microspecies (*G. longipes, G. pubescens, G. burmanicum, G. cardiospermum, G. laxiflorum, G. caulopterum*, and *G. pentagynum*) were extracted from silica-dried leaf material with a simplified CTAB protocol (Doyle, 1987).

**Table 1:** Sampling and assembly information for the eight *Gynostemma* species.

**No**.	**Species**	**Locality**	**Assembly reads**	**Mean length of reads**	**Mean coverage**	**Accession number in GenBank**
**Subgen**. ***Gynostemma***						
1	*G. pentaphyllum*	Xi'an, Shaanxi, China	1,933,796	251.8	3,089.9	KX852298
2	*G. longipes*	Pingli, Shaanxi, China	362,030	151.0	347.1	MF152730
3	*G. pubescens*	Menglun, Kunming, China	780,685	150.7	750.2	MF152732
4	*G. burmanicum*	Menghai, Kunming, China	845,725	150.7	811.3	MF152731
**Subgen**. ***Triostellum***						
5	*G. cardiospermum*	Langao, Shaanxi, China	984,847	149.9	927.6	KX852299
6	*G. laxiflorum*	Xuancheng, Anhui, China	964,649	150.7	921.2	MF136486
7	*G. caulopterum*	Renhuai, Guizhou, China	810,784	150.7	775.2	MF136487
8	*G. pentagynum*	Zhangjiajie, Hunan, China	1,200,521	150.7	1,147.2	KY670737

### Illumina sequencing, assembly, and annotation

Illumina raw reads were generated from an Illumina Hiseq 2500 platform. For *G. pentaphyllum*, the raw reads were assembled using program SPAdes (Bankevich et al., 2012) to obtain the original scaffold. Gapcloser (Luo et al., 2012) and GapFiller (Boetzer and Pirovano, 2012) were used to fill in the gaps and connect the different scaffolds according to the overlap regions. The gaps containing an ambiguous base “N” were validated with PCR amplification productions by designing pairs of primers (Table S1) using Primer3 version 4.0.0 (Untergasser et al., 2012). PrInSeS-G (Massouras et al., 2010) was used to correct the error of bases and indels during assembly. The cp genome was annotated using CpGAVAS (Liu et al., 2012). All the coding DNA sequence (CDS) regions were predicted using EMBOSS (Rice et al., 2000), and then the predicted genes were blasted in eight public databases (NR, CDD, KOG, PFAM, SWISS-PROT, TREMBL, GO, and KEGG) using BLAST with a cut-off e-value of e < 1.0 × 10^−5^, and the similarity degree was set to at least 30%.

Regarding the seven microspecies, the quality-trimmed raw reads were proceeded by CLC Genomics Workbench v7.5 (CLC Bio, Aarhus, Denmark) software with the default parameters set. Reference-guided assembly was performed twice to reconstruct the cp genomes with the program MITObim v1.7 (Hahn et al., 2013) using the obtained *G. pentaphyllum* and published *Cucumis melo* var. *melo* (GenBank accession number: JF412791) as references. A few gaps in the assembled cp genomes were corrected by the Sanger sequencing method (Table S1). The program DOGMA (Wyman et al., 2004) was used to annotate the complete cp genomes, and corrected by comparing with the complete cp genomes of the references mentioned above using GENEIOUS R8 (Biomatters Ltd., Auckland, New Zealand). The circular cp genome maps were drawn using the online program OGDRAW (http://ogdraw.mpimp-golm.mpg.de/).

### Repeated sequences identification

We searched four types of repeated sequences in all eight species. Size and location of dispersed and palindromic repeats were detected using the program REPuter (Kurtz et al., 2001), in which the similarity percentage of two repeat copies was at least 90 % and the parameter of minimal repeat size was 30 bp. The online program Tandem Repeats Finder (http://tandem.bu.edu/trf/trf.html) was used to find the tandem repeat sequences, which was at least 10 bp in length. The alignment parameters for match, mismatch, and indels were set at 2, 7, and 7, respectively. Microsatellites (SSRs) were performed by MISA (Thiel et al., 2003) with thresholds of 10, 5, 4, 3, 3, and 3 for mono-, di-, tri-, tetra-, penta-, and hexa-nucleotide, respectively.

### Sequence divergence analysis

The variable sites across the complete cp genomes, containing LSC, SSC, and IR regions of all species, were computed by DnaSP v5.0 (Librado and Rozas, 2009). MEGA 5.0 (Tamura et al., 2011) was used for statistics on nucleotide substitutions (NSs) and indels of the cp genome sequences to investigate the sequence divergence patterns. The percentage of variable characters for each coding and non-coding region was calculated based on the method of Zhang et al. (2011).

### Whole chloroplast genomes comparison

To discover the interspecific variation among the complete cp genome sequences of eight *Gynostemma* species, we used the program mVISTA (Frazer et al., 2004) to visualize the alignments with annotations using *G. pentaphyllum* as a reference. MAFFT version 7.017 (Katoh and Standley, 2013) was used for multiple alignments of seven microspecies cp genomes with *G. pentaphyllum*. The IR region borders and gene rearrangement among 10 species (eight *Gynostemma* species, the close relative *C. mole* var. *melo*, and the first reported complete cp genome *N. tabacum*) were ascertained by the plug-in program Mauve in GENEIOUS R8 to analyze the expansion variation in junction regions.

### Phylogenetic relationship

For the purpose of reconstructing the phylogenetic relationships and verifying the phylogenetic position of the genus *Gynostemma* in the family Cucurbitaceae, nine published complete cp genome sequences from the orders Cucurbitales and Fagales were also selected in our analyses (Table S2). Sequences were aligned using the program MAFFT version 7.017 (Katoh and Standley, 2013) and manually edited where necessary. Because of the differentiation of molecular evolutionary rates among the different cp genome regions, phylogenetic relationship analyses were performed using the following six datasets: (1) the complete cp genome sequences; (2) 75 common protein coding genes (CDS); (3) one inverted repeats region (IRb); (4) the large single copy region (LSC); (5) the small single copy region (SSC), and (6) the consensus sequences of 10 highly variable regions. The best-fitting model for each dataset based on the Akaike information criterion was determined by Modeltest 3.7 (Posada and Crandall, 1998). Bayesian inference (BI) was implemented with MrBayes 3.12 (Ronquist and Huelsenbeck, 2003) using the settings as following: Markov chain Monte Carlo simulations (MCMC) algorithm for 100,000 generations with four incrementally heated chains, starting from random trees, and sampling one out of every 100 generations. The first 25% of trees were discarded as burn-in (Meng et al., 2008; Ma et al., 2014). The maximum likelihood (ML) trees were reconstructed with RAxML v7.2.8 (Stamatakis, 2006) performed with 1,000 replicates. In all analyses, *Castanea mollissima* and *Castanea pumila* var. *pumila* (Fagaceae) were regarded as outgroups.

## Results

### Gene prediction and genome features

**Figure 1:**
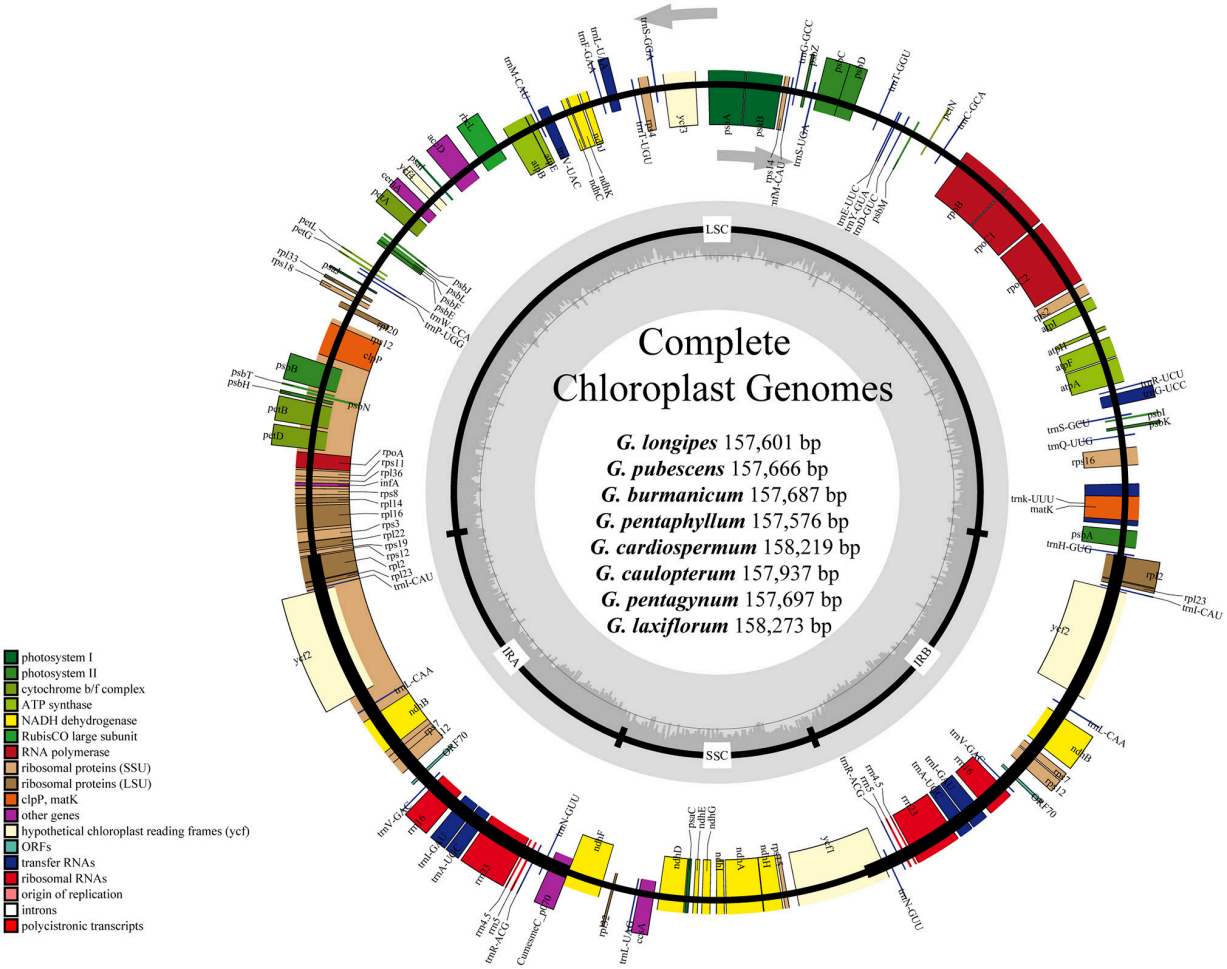
Gene maps of chloroplast genomes of *Gynostemma* BL. Genes on the inside of the large circle are transcribed clockwise and those on the outside are transcribed counterclockwise. The genes are color-coded based on their functions. Dashed area represents the GC composition of the chloroplast genome.

For the cp genome of the widespread *G. pentaphyllum*, 586,299,000 bp reads were produced by Illumina sequencing, including 470,640,574 bp high-quality reads (>Q20), accounting for 80.77% of the total reads with an average length of 251.8 bp. The basic information of sequencing products is shown in Table S3. After preliminary assembly and gene prediction, we obtained a 157,761 bp sequence with four gaps of 176 bp unknown bases, and predicted 91 protein-coding genes, 30 tRNA, and 8 rRNA in total. The predicted protein-coding genes were blasted in the public databases of the known functional genes (Table S4, Figure S1), of which 97.8% could be found in NR databases for species distribution (Figure S2). The top-hit three species, *C. melo* var. *melo, Cucumis sativus*, and *Corynocarpus laevigata*, were identified as belonging to the order Cucurbitales. Moreover, we designed four pairs of primers that successfully amplified the ambiguous regions (Table S1). The initial automatic annotation information of the genome sequence was corrected by comparison with the cp genome of *C. melo* var. *melo* as a reference. Finally, the complete cp genome of *G. pentaphyllum* was fixed to 157,576 bp, which encoded 133 genes, including 87 protein-coding genes, 37 tRNA genes, eight rRNA genes, and one pseudogene (Tables 2, 3, Figure 1).

**Table 2:** Characters of Illumina sequencing and assembly of chloroplast genome of eight *Gynostemma* species.

**Feature**	***G. pentaphyllum***	***G. longipes***	***G. pubescens***	***G. burmanicum***	***G. cardiospermum***	***G. laxiflorum***	***G. caulopterum***	***G. pentagynum***	**Average value**
Size (bp)	157,576	157,601	157,666	157,687	158,219	158,273	157,937	157,697	157,832
LSC (bp)	86,757	86,780	86,811	86,835	87,110	87,047	86,846	86,489	86,834
SSC (bp)	18,653	18,647	18,627	18,624	18,627	18,708	18,583	18,724	18,649
IRs (bp)	26,083	26,087	26,114	26,114	26,241	26,259	26,254	26,242	26,174
Total genes	133	133	133	133	133	133	133	133	133
Protein-coding genes	87 (7)	87 (7)	87 (7)	87 (7)	87 (7)	87 (7)	87 (7)	87 (7)	87 (7)
tRNA genes	37 (7)	37 (7)	37 (7)	37 (7)	37 (7)	37 (7)	37 (7)	37 (7)	37 (7)
rRNA genes	8 (4)	8 (4)	8 (4)	8 (4)	8 (4)	8 (4)	8 (4)	8 (4)	8 (4)
Overall GC content (%)	37	37	37	37	36.9	37	37	37	36.9875
GC content in LSC (%)	34.8	34.8	34.9	34.9	34.8	34.8	34.8	34.8	34.825
GC content in SSC (%)	30.6	30.6	30.7	30.7	30.7	30.7	30.8	30.6	30.675
GC content in IR (%)	42.8	42.8	42.8	42.8	42.8	42.8	42.8	42.8	42.8

*The numbers in brackets indicate the genes duplicated in the IR regions*.

**Table 3:** List of genes in the chloroplast genome of eight *Gynostemma* BL. species.

**Group of genes**	**Name of genes**
Photosystem I (5)	*psaA psaB psaC psaI psaJ*
Photosystem II (15)	*psbA psbB psbC psbD psbE psbF psbH psbI psbJ psbK psbL psbM psbN psbT psbZ*
Cytochrome b/f complex (6)	*petA*^a^*petB*^a^*petD petG petL petN*
ATP synthase (6)	*atpA atpB atpE*^a^*atpF atpH atpI*
NADH dehydrogenase (12)	^a^*ndhA*^a^*ndhB (*×2) *ndhC ndhD ndhE ndhF ndhG ndhH ndhI ndhJ ndhK*
Rubisco large subunit (1)	*rbcL*
Ribosomal protein (small subunit) (14)	*rps2 rps3 rps4 rps7 (*×2) *rps8 rps11*^b^*rps12 (*×2) *rps14 rps15*^a^*rps16 rps18 rps19*
Ribosomal protein (large subunit) (11)	^b^*rpl2 (*×2) *rpl14*^a^*rpl16 rpl20 rpl22 rpl23 (*×2) *rpl32 rpl33 rpl36*
RNA polymerase (4)	*rpoA rpoB*^a^*rpoC1 rpoC2*
membrane protein (1)	*cemA*
Acetyl-CoA carboxylase gene (1)	*accD*
ATP-dependent protease subunit (1)	^b^*clpP*
Assembly/stability of photosystem I (2)	^b^*ycf3 ycf4*
Maturase (1)	*matK*
Conserved reading frames (ycfs) (4)	*ycf1* (×2) *ycf2* (×2)
c-type Cytochrome biogenesis (1)	*ccsA*
Translation-related gene (1)	^P^*infA*
Transfer RNAs (37)	^a^*trnA-UGC (*×2) *trnC-GCA trnD-GUC trnE-UUC trnF-GAA trnfM-CAU*^a^*trnG-UCC trnG-GCC trnH-GUG trnI-CAU* (×2)^a^*trnI-GAU (*×2)^a^*trnK-UUU trnL-CAA* (×2) *trnL-UAG*^a^*trnL-UAA trnM-CAU trnN-GUU* (×2) *trnP-UGG trnQ-UUG trnR-ACG* (×2) *trnR-UCU trnS-GCU trnS-GGA trnS-UGA trnT-GGU trnT-UGU trnV-GAC* (×2)^a^*trnV-UAC trnW-CCA trnY-GUA*
Ribosomal RNAs (8)	*rrn4.5* (×2) *rrn5* (×2) *rrn16* (×2) *rrn23* (×2)
hypothetical chloroplast protein (2)	*orf70* (×2)

a *Gene with one intron*.

b *Gene with two introns*.

P *Pseudogene; (×2): Gene with two copies*.

The consensus sequences of the seven cp genomes of the microspecies were assembled by mapping each Illumina raw read to the reference genome sequences. The seven microspecies cp genomes ranged from 157,601 bp (*G. longipes*) to 158,273 bp (*G. laxiflorum*), and the average length was 157,832 bp. All reads and coverage of the cp genomes are displayed in Table 1. The gene number and varieties were consistent with *G. pentaphyllum* (Table 2, Figure 1). The annotated circular cp genome sequences were submitted to the GenBank and accession numbers were obtained (Table 1).

**Figure 2:**
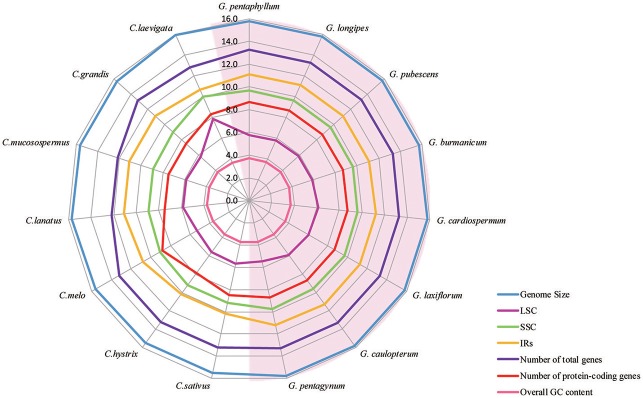
A radar-plot comparing features of the complete chloroplast genomes of 15 accessions of Cucurbitales species. Showing, from inside to out, overall GC content, LSC region size, number of protein-coding genes, SSC region size, IR region size, number of total genes and genome size.

All of the cp genomes displayed a typical quadripartite structure, two copies of inverted repeats (IRs, 26,174 bp average) segregated by two SC regions, namely a large single copy region (LSC, 86,834 bp average) and small single copy region (SSC, 18,649 bp average, Table 2). Comparison of cp genome sequences among eight *Gynostemma* species and seven other Cucurbitales plants (Table S5) showed no dramatic difference in compared features (Figure 2). The GC content percentage of *G. cardiospermum* (36.9%) was less than any other genome (37.0%), whereas the GC content of the IR region was clearly higher than that of any other region of each cp genome (Table 2). The cp genomes encoded an identical set of 133 functional genes, of which 18 were duplicated in the IR region, including seven protein-coding genes, seven transfer RNA (tRNA) genes, and four ribosomal RNA (rRNA) genes. Twenty-three genes had introns, four of them (two *rps12*, one *clpP* and one *ycf3*) with two introns. The gene *infA*, which was a translation-related gene, was identified as a pseudogene (Table 3).

### Repeat analysis and simple sequence repeats (SSR)

**Figure 3:**
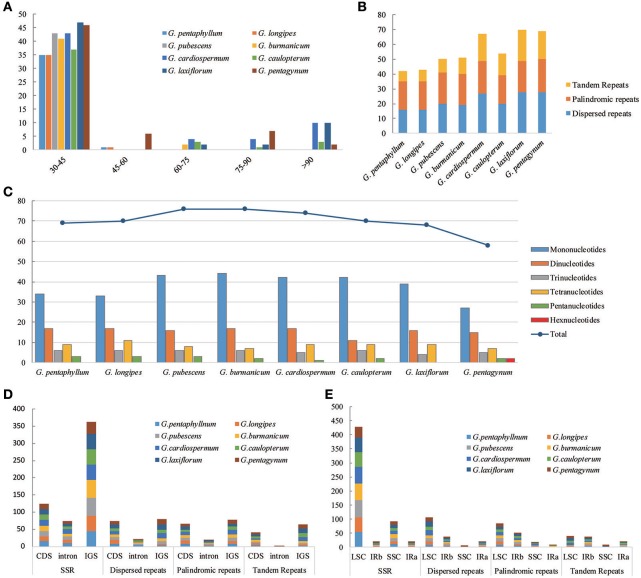
The distribution, type and presence of repeated sequences and simple sequence repeats (SSR) in the cp genome of eight *Gynostemma* species. **(A)** Number of repeat sequences by length; **(B)** Number of three repeat types in the eight chloroplast genomes; **(C)** Number of SSR types in the eight chloroplast genomes; **(D,E)** Location of the all repeats from eight species.

The types and distribution of repeated sequences and the presence of SSRs were analyzed in the cp genomes of eight *Gynostemma* species. Sixteen dispersed repeats, 19 palindromic repeats, and seven tandem repeats were discovered in the *G. pentaphyllum* cp genome. In the *G. cardiospermum* cp genome, the numbers of these three types of repeated sequences were 27, 22, and 18, respectively. The repeat number of the other six microspecies are shown in Figure 3B. Repeated sequences of Subgen. *Triostellum* species were much greater than those of Subgen. *Gynostemma* species. Most repeats (73.32%) were 30–45 bp in length (Figure 3A). The repeated sequences were mostly distributed in the intergenic spacers (IGS) and intron regions (Figure 3D), but some repeats were also found in coding regions (CDS), such as *ycf2, psaB*, and *trnS*, among others (Tables S6, S7). Moreover, SSRs of the eight cp genomes were analyzed (Figure 3C). Among them, SSRs of *G. pubescens* and *G. burmanicum* (77) were the greatest and that of *G. pentagynum* (58) were the lowest. In total, 561 SSRs were discovered, of which 77.76% were distributed in the CNS and 125 SSRs were found in the CDS (Figure 3D, Table S8). For the four structural regions in cp genomes, 429 SSRs were found in LSC, whereas 20, 92, and 20 were in IRb, SSC, and IRa, respectively (Figure 3E). It appeared that the SSRs were distributed unevenly across the cp genomes. There were 304, 126, 44, 69, and 16 mono-, di-, tri-, tetra-, and penta-nucleotide repeats, respectively. It was noteworthy that *G. pentagynum* had two hexanucleotides SSRs (Figure 3C). Among these SSRs, mononucleotide repeats were common and accounted for 54.19% of the total, whereas dinucleotides accounted for 22.46%, and the other polynucleotide SSRs occurred with less frequency (Figure 3C).

### Sequence divergence

**Figure 4:**
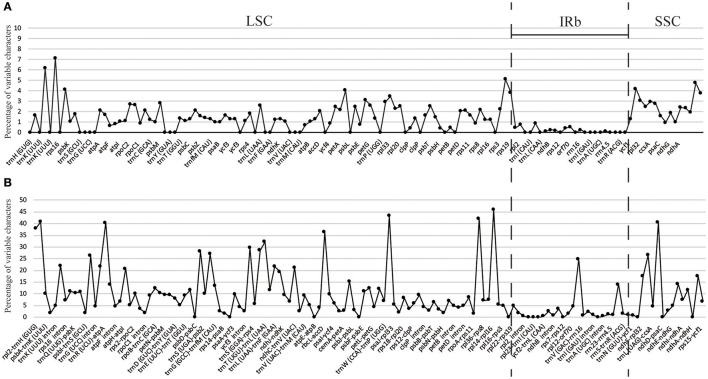
Percentage of variable characters in aligned eight *Gynostemma* chloroplast genomes. **(A)** Coding region (CDS) and **(B)** Noncoding region (CNS); These regions are oriented according to their locations in the chloroplast genome.

The percentage of variation for eight *Gynostemma* cp genomes in the CNS ranged from 0 to 45.98%, with a total of 10.86%, which was much higher than that in the CDS (0–7.14, 2.11% in total, Table 4, Figure 4), indicating that the CDS were much more conservative than the CNS. Furthermore, the mean percentage of variation in IRs (2.59 and 0.51%) was lower than that of LSC (12.52 and 1.93%) or SSC (16.84 and 4.13%), which demonstrated that the IR region had fewer mutations and was highly conserved. The genes *rps16, matK, rpl22, rps15*, and *ndhF* were the top five genes exhibiting higher variability (variation percentage ≥4.21%, Figure 4A) than other genes. It was notable that gene *ndhD* in *G. caulopterum* was 144 bp shorter than others because of an indel of AT in the initiation region (Figure S3). We further analyzed the sequence divergence patterns in all of the cp genomes. Finally, 3,425 nucleotide substitution (NS) loci with 3,885 bp and 1,116 indel loci with 4,750 bp were found in the aligned sequence.

**Table 4:** Variable sites analyses of eight *Gynostemma* chloroplast genomes.

	**Length (bp)**	**Number of variable sites**	**Number of variable bases (bp)**	**Mean percentage of variability**
**Coding region**	73,268	1264	1543	2.106 (0.000–7.143)
LSC	44,837	774	865	1.929 (0.000–7.143)
IR	13,666	65	69	0.505 (0.000–1.314)
SSC	14,765	425	609	4.125 (0.980–4.762)
**Noncoding region**	61,677	3114	6695	10.855 (0.000–45.977)
LSC	43,709	2593	5471	12.517 (0.000–45.977)
IR	12,646	98	328	2.594 (0.000–24.784)
SSC	5,322	423	896	16.836 (0.000–40.446)

*Values in brackets were the range of variation percentages in each region*.

### Comparison of border regions and sequence identity

**Figure 5:**
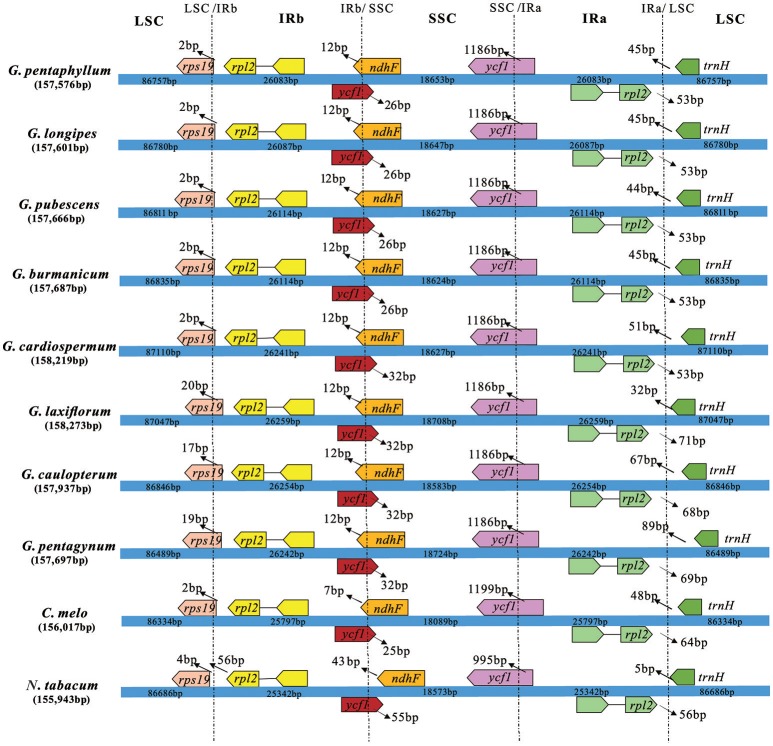
Comparison of the border positions of LSC, SSC, and IR regions in four chloroplast genomes. Boxes above or below the main line represent the genes at the IR/SC borders.

The border regions of the eight *Gynostemma* cp genomes were compared with *C. melo* var. *melo* and *N. tabacum* to analyze the expansion and contraction variation in junction regions. The IR regions of 10 cp genomes ranged from 25,342 bp (*N. tabacum*) to 26,259 bp (*G. laxiflorum*) in size, of which *rps19, ycf1, ndhF, rpl2*, and *trnH* genes were present at the junctions of the LSC/IR and SSC/IR borders. Considerable variation was observed in the expansion and contraction of IR regions (Figure 5). For the LSC/IR borders, the gene *rps19* in the LSC of *Gynostemma* species extended 2–20 bp into the IRb region, whereas *N. tabacum* contracted four bp instead. The gene *trnH* in the LSC region contracted 32–89 bp from the junction region of IRa/LSC. The gene *rpl2* in the IRa region also contracted by a different number of bases (53–71 bp). In contrast, the SSC/IR boundary regions were relatively stable. The gene *ycf1* in the IRb region and gene *ndhF* in the SSC region interlaced at the IRb/SSC border, and *ycf1* in the SSC region was astride the border of SSC/IRa. Gene *ndhF* and *ycf1* in the SSC region extended the same number of bases among the eight *Gynostemma* species (12 and 1,186 bp, respectively), whereas gene *ycf1* in the IRb region exhibited an interesting expansion: Subgen. *Gynostemma* species extended 26 bp into the SSC region, whereas the Subgen. *Triostellum* species extended 32 bp instead. We also compared the cp genomes of 10 species mentioned above to identify genome rearrangement events for each species. It was shown that no rearrangement events occurred in the eight *Gynostemma* plants (Figure S4).

**Figure 6:**
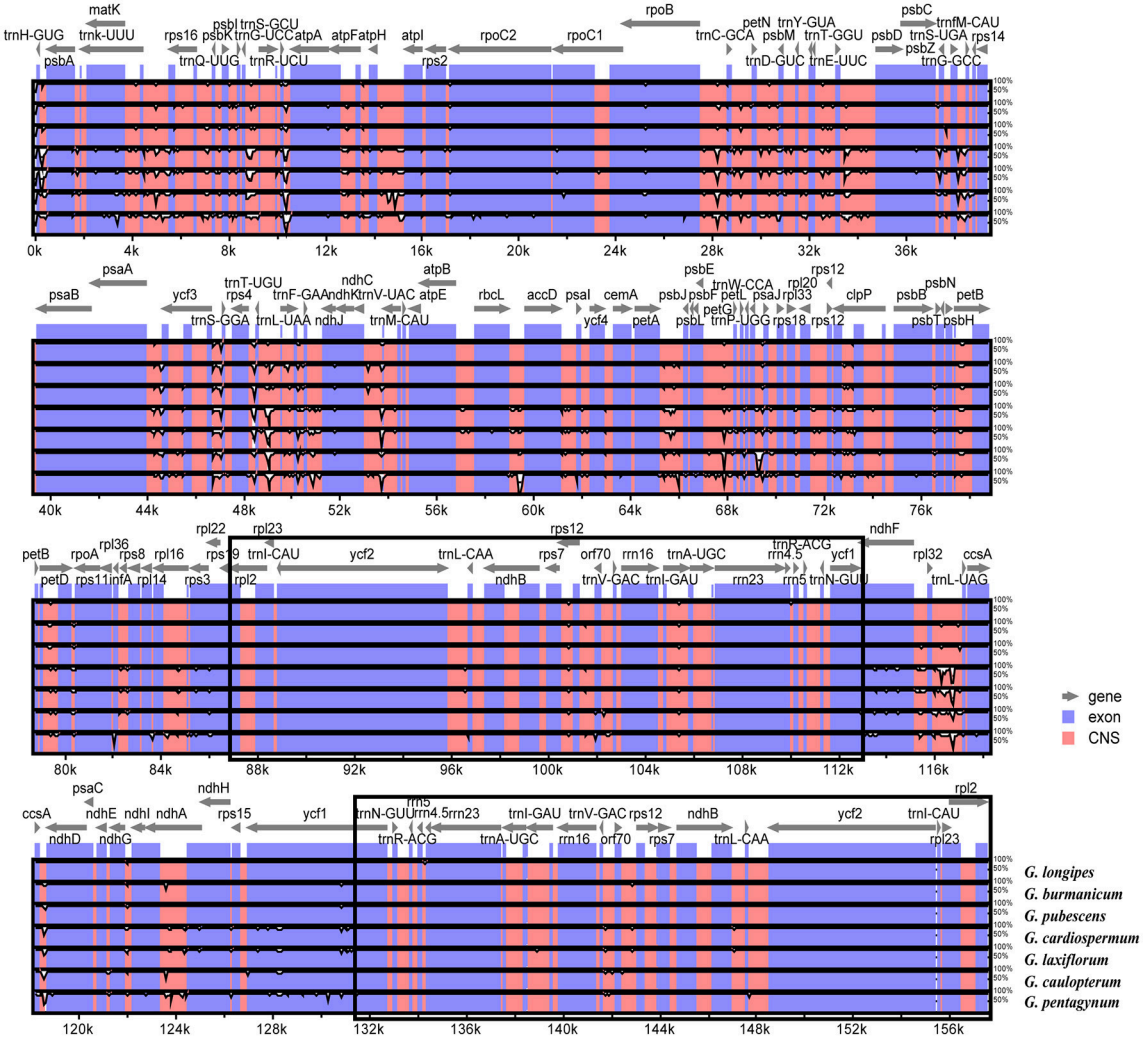
Sequence identity plots among eight sequenced chloroplast genomes, with *G. pentaphyllum* as a reference by using mVISTA. The vertical scale indicates the identity percentage ranging from 50 to 100%. The horizontal axis corresponds to the coordinates within the chloroplast genome. Coding and non-coding regions are marked in blue and pink, respectively. Annotated genes are displayed along the top. The black boxes show the two IR regions.

The overall sequence identity of eight *Gynostemma* cp genomes were performed with *G. pentaphyllum* as a reference (Figure 6). The alignment showed high sequence similarity. However, most of the significant divergence was found in the CNS. Among them, a high degree of divergence included *ycf3-trnS (GGA), trnT (UGU)-trnL (UAA), rpl32-trnL (UAG), ccsA-ndhD*, and *petA-psbJ*. The most similar sequences were in four rRNA genes, photosystem I genes, cytochrome b/f complexes, and ATP synthase genes. As previously mentioned, the IR regions exhibited lower sequence divergence than did the SC regions, suggesting that they were highly conserved.

### Phylogenetic analysis

**Figure 7:**
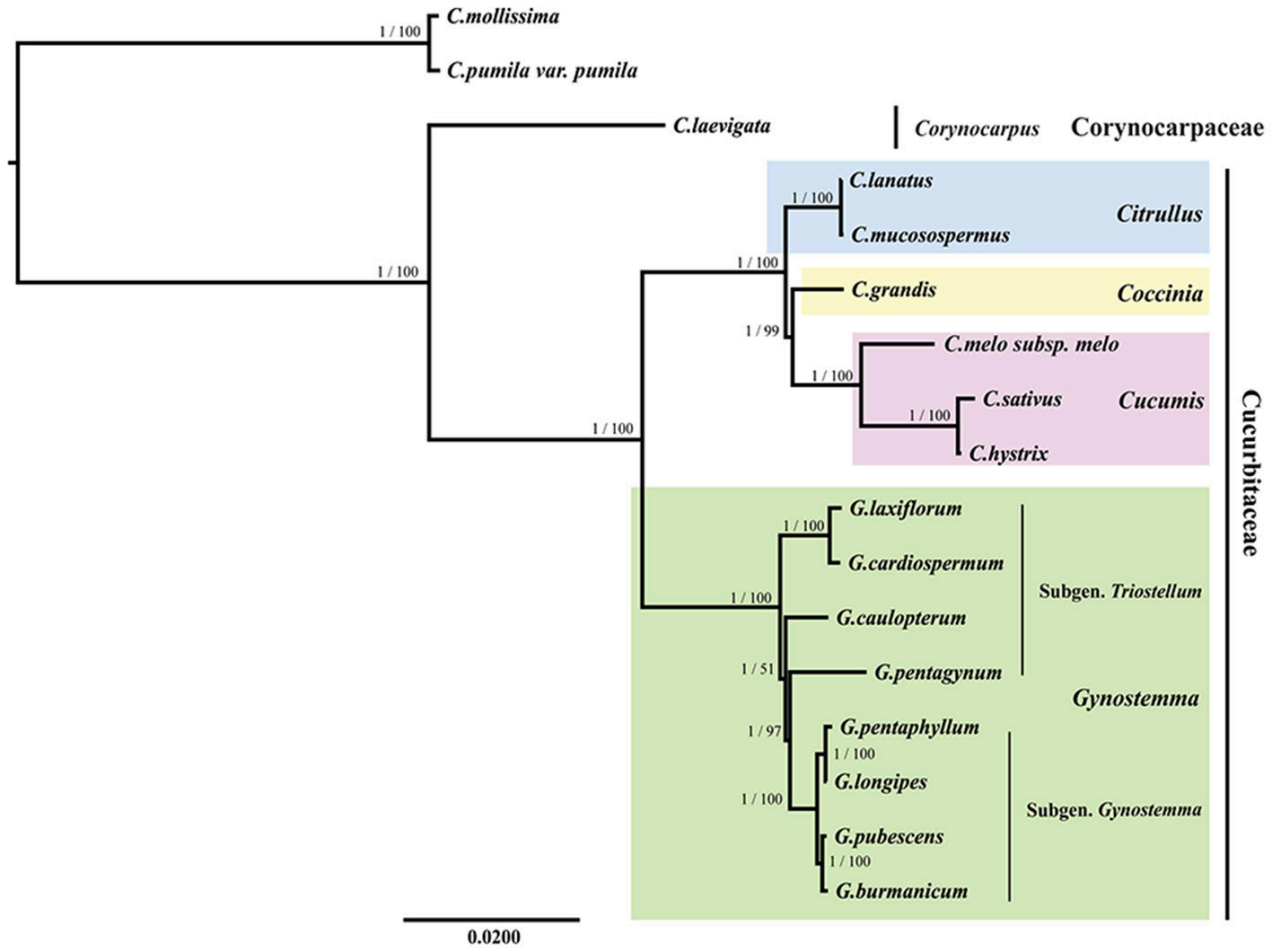
Phylogenetic relationship of the 17 species inferred from BI and ML analyses based on the complete cp genome sequences. The Bayesian posterior probabilities and bootstrap values of ML analyses are shown beside the clades. *Castanea mollissima* and *Castanea pumila* var. *pumila* were used as the outgroups.

All the BI and ML trees reconstructed based on the six datasets were congruent in identification of the phylogenetic position of the genus *Gynostemma* in the family Cucurbitaceae. The best-fit models for each dataset used in BI and ML analysis are displayed in Table 5. The phylogeny produced from the analysis of 17 complete cp genome sequences was well-supported. All nodes of the phylogenetic trees were strongly supported by 1.00 Bayesian posterior probabilities in BI analysis and 51–100% bootstrap values in ML analysis (Figure 7). It was shown that *C. laevigata* was the earliest diverging lineage in this group, which was identified as a sister to other species. All species of the Subgen. *Gynostemma* (*G. pentaphyllum, G. longipes, G. pubescens*, and *G. burmanicum*) and Subgen. *Triostellum* (*G. cardiospermum, G. laxiflorum, G. caulopterum*, and *G. pentagynum*) formed a *Gynostemma* clade. Combined with the species of the genera *Cucumis, Coccinia*, and *Citrullus*, indicated the family Cucurbitaceae was monophyletic. The phylogenetic trees of other datasets also showed a similar and clear internal relationship of Cucurbitales plants (Figure S5).

**Table 5:** Best-fit Models in ML and BI analysis.

**Datasets**	**Model in ML**	**Model in BI**
ALL	GTR+I+G	TVM+I+G
LSC	GTR+I+G	TVM+I+G
IR	GTR+I+G	GTR+G
SSC	GTR+I+G	TVM+G
CDS	GTR+I+G	GTR+I+G
10 highly variable regions	GTR+I+G	TVM+G

## Discussion

### Chloroplast sequence variation and evolution

We determined the complete nucleotide sequences of eight *Gynostemma* cp genomes using Illumina paired-end sequencing combined with the Sanger sequencing method. Because no perfect assembler program has been created, *de novo* assembly always generates misassembled contigs; thus, assembled contigs must be checked and corrected by read-mapping and further scanned for any gaps of lower coverage (Naito et al., 2013; Nashima et al., 2015). Despite the occurrence of gene loss-and-gain events and the rearrangements in several genomes of land plants (Palmer, 1987; Fu et al., 2016), the eight cp genomes we studied displayed the typical quadripartite structure with two IRs and two SCs. In terms of gene content, each of the cp genomes encoded 133 genes containing with 87 protein-coding genes, 37 tRNA genes, eight rRNA genes, and one pseudogene. The function, order, and GC-content of these genes were all highly conserved as noted for other angiosperms (Palmer, 1991). The cp genomes of all the *Gynostemma* species contained more AT and had a GC content of 36.9–37.0%, similar to previously published *Olea* and *Diospyro*s genomes (Mariotti et al., 2010; Fu et al., 2016), and possibly caused by the high GC content of the rRNA gene sequences located in IR regions.

Pseudogenes are functionless relatives of genes that have lost their ability to code a protein (Vanin, 1985), and are generally regarded as the last stop for genomic material and are often thought to be “junk DNA” (Zheng et al., 2007). Nonetheless, recent research suggested that pseudogenes were evolutionary relics of functional components in the genome that provide important information about the history of the gene and genome evolution (Balakirev and Ayala, 2003; Zou et al., 2009). Although not protein-coding, the DNA of pseudogenes may be similar to other kinds of non-coding DNA, which may have a regulatory function (Poliseno et al., 2010). In this study, gene *infA*, which is located between genes *rpl36* and *rps8* in the LSC region was identified as a pseudogene because of the presence of several internal stop codons. The gene *infA* acts as a transcription anti-terminator and has RNA chaperone activity (Phadtare et al., 2007). It also exists as a pseudogene in many other cp genomes [e.g., *Syzygium cumini* and *Ananas comosus* (Asif et al., 2013; Nashima et al., 2015)], or is lost [*Arabidopsis thaliana* and *Alstroemeria aurea* (Sato et al., 1999; Do et al., 2013)]. However, many other pseudogenes, for instance, the *ycf15* gene in *Quercus spinose* (Du et al., 2015), *rpl22* and *rps18* genes in the Paeoniaceae (Dong et al., 2013) were predicted to be lost or normal in our study.

Previous research supports that repeated sequences may play an important role in rearranging sequences and producing variation that cp genomes lost through slipped-strand mispairing and illegitimate recombination (Cavalier Smith, 2002). The presence of dispersed, palindromic, and tandem repeats in *Gynostemma* cp genomes were reported. The repeats in Subgen. *Triostellum* species were greater than those in Subgen. *Gynostemma* species. The majority were distributed in the IGS and intron regions, which were the highly variable regions in the cp genomes. The cp SSRs have been detected in *Oryza sativa* (Ishii and McCouch, 2000), *Haloxylon ammodendron*, and *Haloxylon persicum* (Dong et al., 2016). We also identified 561 SSRs within eight species, 77.72 % of them were distributed in the IGS and intron regions. Overall, the SSRs can be used to analyze the population genetics and evolutionary studies based on their polymorphism leading to sensitive genetic diversity, population structure, and phylogeographic studies at the inter- and intrapopulation levels (Pauwels et al., 2012; Naydenov et al., 2016). Thus, *Gynostemma* cp SSRs could contribute to evolutionary and molecular ecological knowledge, which warrants further research.

Although the extant studies point that the cp genome of herbaceous plants has been evolving rapidly (Zhong et al., 2009) and has several structural changes such as gene inversion (Doyle et al., 1992) and gene loss-and-gain events (Diekmann et al., 2009), no rearrangement events were found in all of our species after global alignment with the published cp genomes of *C. melo* var. *melo*, and *N. tabacum*. The cp genomes are highly conserved in terms of size and genomic structure, whereas usually it is different among species within a family (Sun et al., 2013). In addition, the IR regions are important for stabilizing cp genome structure (Maréchal and Brisson, 2010). IR regions are highly conserved, but our results indicated that some position changes occurred in the IR/SC border areas. They may have been caused by the contraction and expansion events of the IR region, which was mainly responsible for length mutations of cp genomes and is a common evolutionary phenomenon in plants (Kim and Lee, 2004). However, borders between two regions among the eight *Gynostemma* species showed high similarity, especially for gene *ndhF* located in the SSC region and *ycf1* in the IRb region located in the LSC. It was interesting that the gene positions for Subgen. *Gynostemma* species were stable but for those of Subgen. *Triostellum* species were variable and differed from one another.

The sequence identity revealed low differentiation among species within a single subgenus, but great differences between the cp genomes of two subgenera. As expected, in conformity with most angiosperms, IRs and CDS were more conserved than SCs and CNS. This was consistent with the result of sequence divergence where the mean percentage of variations in IRs was lower than that of the SCs. Remarkably, the top five genes exhibiting higher variability and the high-divergence regions, which were always identified as hotspots, had been described. Therefore, further work on developing universal primers and candidate DNA barcodes for these regions would be necessary to judge whether it is conducive to the assessment of the phylogenetic relationships among *Gynostemma* species.

### Phylogenetic analysis

There has been an increasing number of studies using complete cp genome sequences for assessing phylogenetic relationships among angiosperms (Bock et al., 2014; Raman et al., 2016). Our phylogenetic trees indicated a very clear internal relationship of restricted Cucurbitales plants with high bootstrap values. The phylogenetic trees showed that the eight species of *Gynostemma* clustered into Cucurbitaceae and paralleled the sister taxa of *Cucumis, Coccinia*, and *Citrullus* species. This is consistent with the phylogenetic position of the genus *Gynostemma* in the morphological classification by Chen (Chen, 1995). However, our study was just a glimpse of phylogenetic relationships for the species of *Gynostemma*, we will estimate and discuss the interior phylogenetic relationships within the genus *Gynostemma* with more comprehensive and in-depth analyses, and enhance our profound understanding of the systematic evolution of Cucurbitaceae in the future.

## Author contributions

GZ and XZ conceived and designed the experiment. XZ and TZ performed the experiments and analyzed the data. YZ and GB collected the samples. XZ wrote the paper. TZ and NK helped to revise the paper. All authors read and approved the final manuscript.

### Conflict of interest statement

The authors declare that the research was conducted in the absence of any commercial or financial relationships that could be construed as a potential conflict of interest.

## Supplementary material

Table S1 Primers for low coverage regions of eight *Gynostemma* species.

Table S2 List of species and their accession numbers in GenBank included in the phylogenetic analyses of complete chloroplast genomes.

Table S3 Basic Information of Illumina Paired-end Sequencing Products of *G. pentaphyllum*.

Table S4 The result of comparison for predicted protein-coding genes and the public databases.

Table S5 Characteristics of chloroplast genomes of 15 different accessions of Cucurbitales species.

Table S6 List of tandem repeats in the chloroplast genome of eight *Gynostemma* species.

Table S7 List of dispersed repeats and palindromic repeats in the chloroplast genome of eight *Gynostemma* species.

Table S8 List of SSRs in the chloroplast genome of eight *Gynostemma* species.

Figure S1 Percentage of annotated genes in eight public databases.

Figure S2 Species distribution of predicted protein-coding genes of *G. pentaphyllum* in NR databases.

Figure S3 Alignment of gene *ndhD* in eight *Gynostemma* species. The purple box with a letter M presents initiation codons.

Figure S4 Genome rearrangement events of eight *Gynostemma* species, comparing with *C. melo* var. *melo* and *N. tabacum*.

Figure S5 Phylogenetic relationship of the 17 species inferred from BI and ML analyses based on five datasets (A. CDS; B. IR; C. LSC; D. SSC, and E. consensus sequences of 10 highly variable regions). The Bayesian posterior probabilities and bootstrap values of ML analyses are shown beside the clades. *Castanea mollissima* and *Castanea pumila* var. *pumila* were used as the outgroups.
