# Supplementary figures and images for: Completion of Eight Gynostemma BL. (Cucurbitaceae) Chloroplast Genomes: Characterization, Comparative Analysis, and Phylogenetic Relationships
Source: Front Plant Sci. 2017 Sep 12;8:1583. doi: 10.3389/fpls.2017.01583 (PMC5600969; doi:10.3389/fpls.2017.01583)

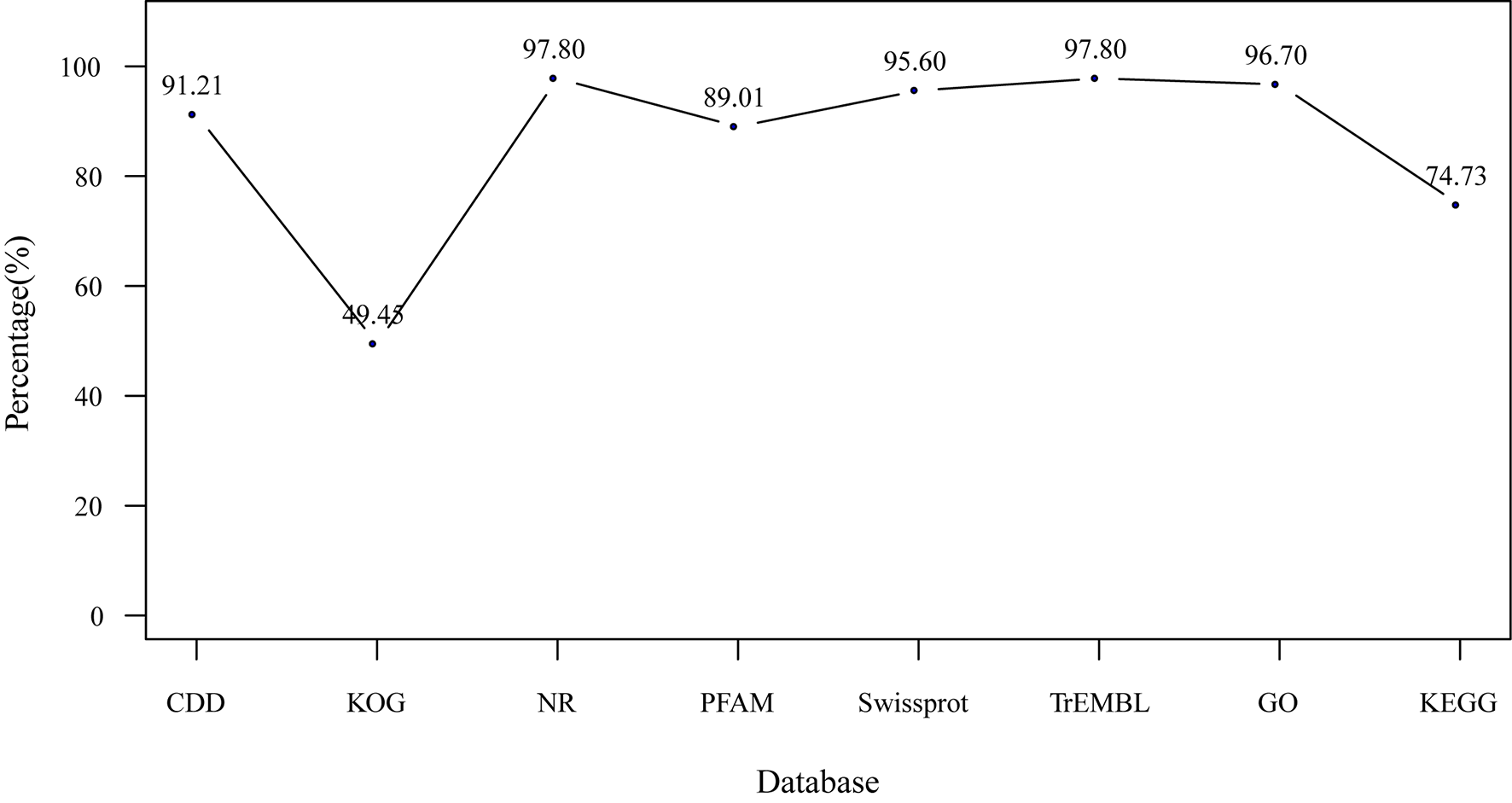

Supplement: Figure S1 — Percentage of annotated genes in eight public databases. [file Image1.TIF]

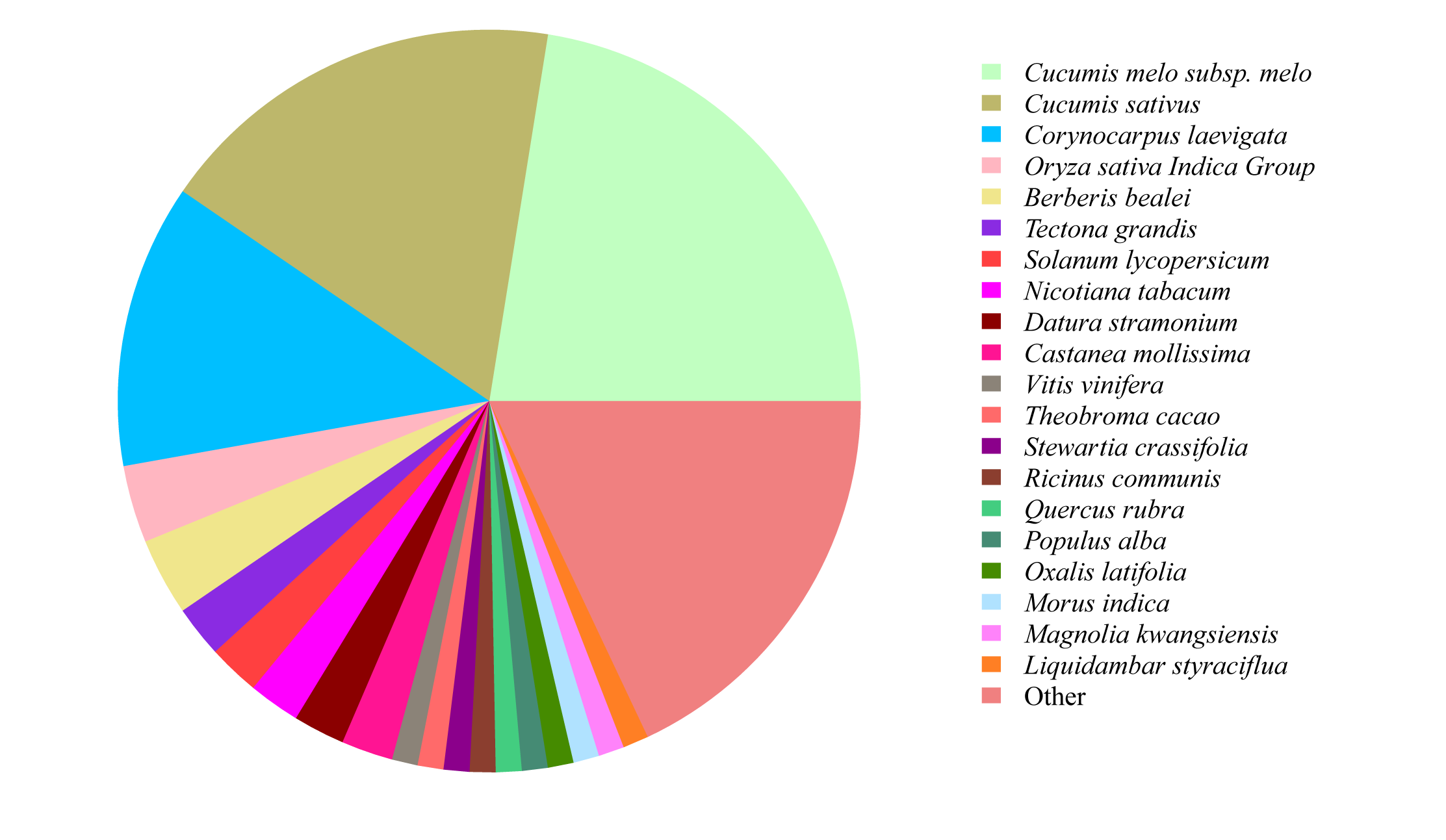

Supplement: Figure S2 — Species distribution of predicted protein-coding genes of G. pentaphyllum in NR databases. [file Image2.TIF]

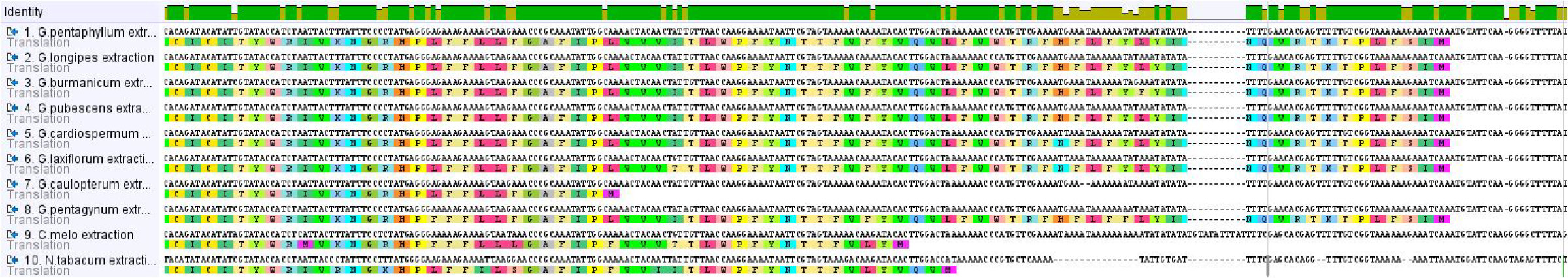

Supplement: Figure S3 — Alignment of gene ndhD in eight Gynostemma species. The purple box with a letter M presents initiation codons. [file Image3.TIF]

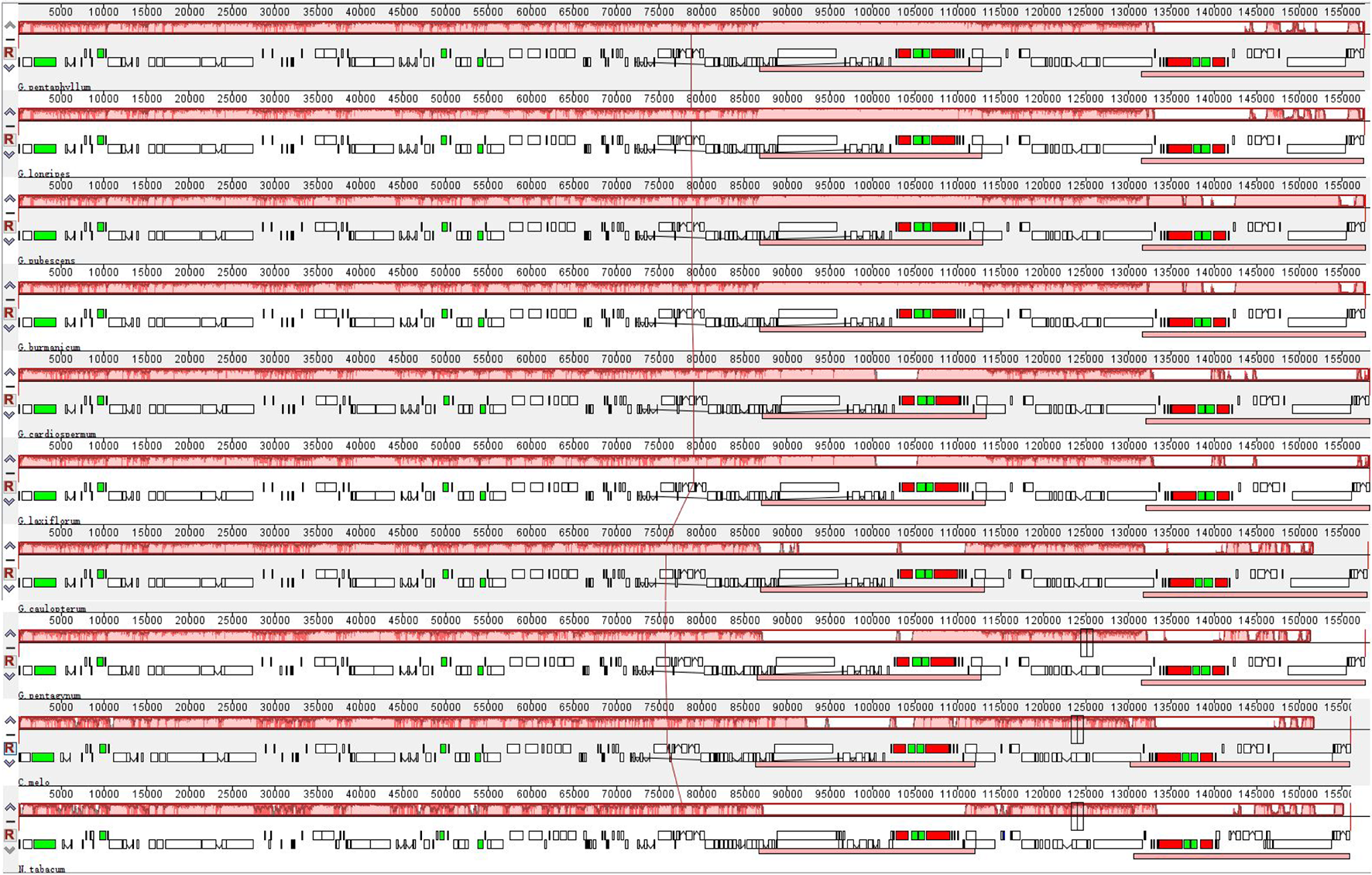

Supplement: Figure S4 — Genome rearrangement events of eight Gynostemma species, comparing with C. melo var. melo and N. tabacum. [file Image4.TIF]

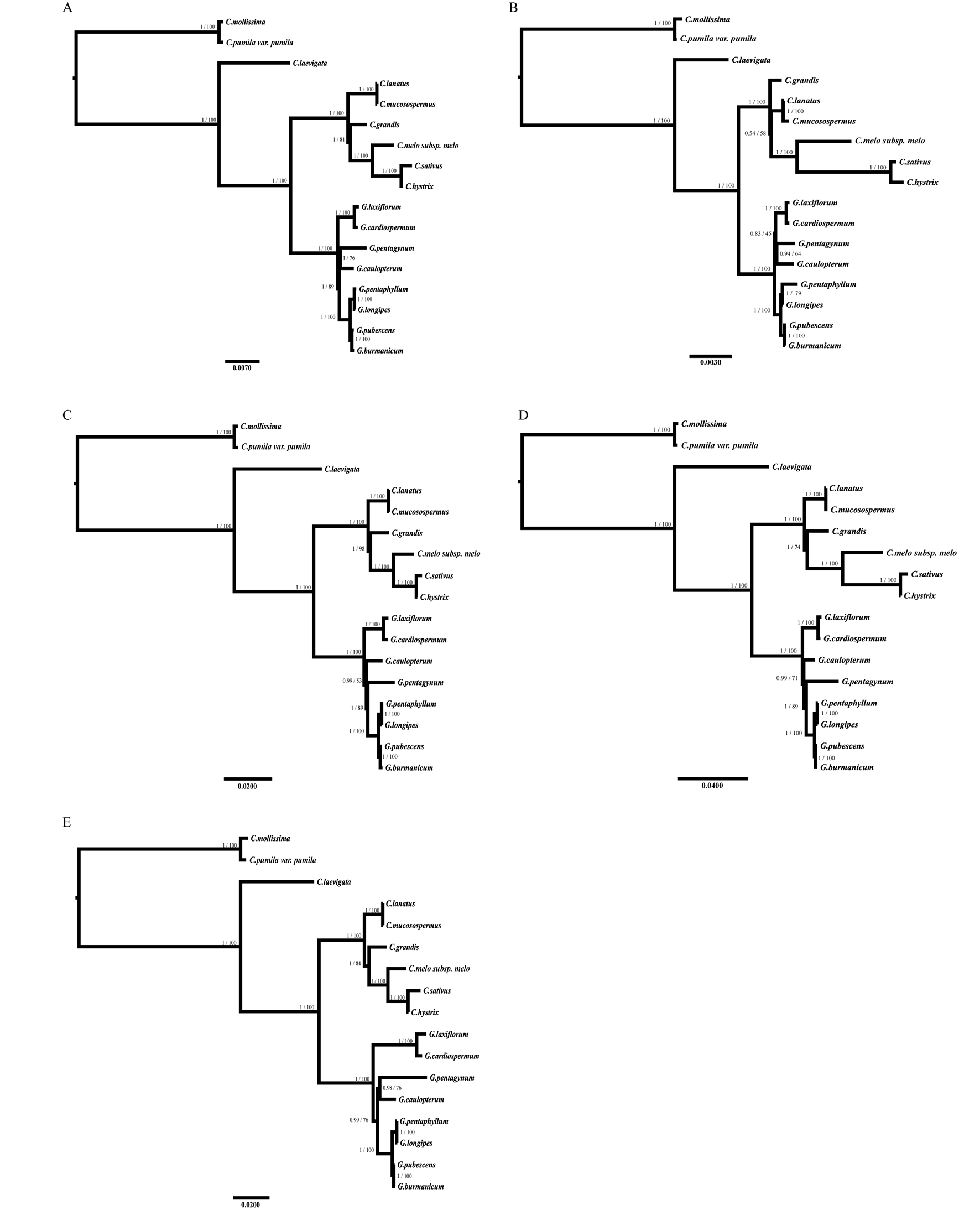

Supplement: Figure S5 — Phylogenetic relationship of the 17 species inferred from BI and ML analyses based on five datasets (A. CDS; B. IR; C. LSC; D. SSC, and E. consensus sequences of 10 highly variable regions). The Bayesian posterior probabilities and bootstrap values of ML analyses are shown beside the clades. Castanea mollissima and Castanea pumila var. pumila were used as the outgroups. [file Image5.TIF]
